# Unsuitability of the Oxidation-Reduction Potential Measurement for the Quantification of Fecal Redox Status in Inflammatory Bowel Disease

**DOI:** 10.3390/biomedicines11123107

**Published:** 2023-11-21

**Authors:** Sem Geertsema, Bernadien H. Jansen, Harry van Goor, Gerard Dijkstra, Klaas Nico Faber, Arno R. Bourgonje

**Affiliations:** 1 Department of Gastroenterology and Hepatology, University of Groningen, University Medical Center Groningen, 9713 GZ Groningen, The Netherlands; s.geertsema@umcg.nl (S.G.); b.h.jansen@umcg.nl (B.H.J.); gerard.dijkstra@umcg.nl (G.D.); k.n.faber@umcg.nl (K.N.F.); 2 Department of Pathology and Medical Biology, University of Groningen, University Medical Center Groningen, 9713 GZ Groningen, The Netherlands; h.van.goor@umcg.nl; 3 The Henry D. Janowitz Division of Gastroenterology, Department of Medicine, Icahn School of Medicine at Mount Sinai, New York, NY 10029, USA

**Keywords:** Crohn’s disease (CD), ulcerative colitis (UC), oxidative stress, electrochemical method, proof-of-concept study

## Abstract

Oxidative stress is a key pathophysiological process associated with the development and progression of inflammatory bowel disease (IBD). Biomarkers for oxidative stress, however, are scarce, as are diagnostic tools that can interrogate an individual’s gut redox status. This proof-of-concept study aimed to evaluate the potential utility of an oxidation-reduction potential (ORP) measurement probe, to quantify redox status in the feces of both patients with IBD and healthy controls. Previous studies using this ORP measurement probe demonstrated promising data when comparing ORP from severely malnourished individuals with that of healthy controls. To date, ORP analyses have not been performed in the context of IBD. We hypothesized that measuring the ORP of fecal water in patients with IBD might have diagnostic value. The current study, however, did not show significant differences in ORP measurement values between patients with IBD (median [IQR] 46.5 [33.0–61.2] mV) and healthy controls (25 [8.0–52.0] mV; *p* = 0.221). Additionally, ORP measurements were highly unstable and rapidly fluctuated throughout time, with ORP values varying from +24 to +303 mV. Due to potential biological processes and limitations of the measuring equipment, this study was unable to reliably measure ORP. As a result, our findings indicate that ORP quantification may not be a suitable method for assessing fecal redox status and, therefore, does not currently support further exploration as a diagnostic or monitoring tool.

## 1. Introduction

Inflammatory bowel disease (IBD), encompassing Crohn’s disease (CD) and ulcerative colitis (UC), is a chronic and debilitating disease characterized by relapsing inflammation of the gastrointestinal tract [1]. The pathogenesis of IBD is complex, involving a combination of genetic, environmental, dietary, and microbial factors [2]. These elements all interact with each other and contribute to IBD development and progression, resulting in disturbed gut mucosal homeostasis and distinct immunological alterations [2].

Oxidative stress is commonly defined as an imbalance between oxidants and antioxidants in favor of the oxidants, leading to a disruption of redox signaling and control, and/or molecular damage [3]. Oxidative stress is mainly characterized by an overproduction of reactive species, including reactive oxygen species (ROS), reactive nitrogen species (RNS), and reactive sulfur species (RSS) [4,5,6]. Specifically, ROS originate mostly from the mitochondria, inside which specific enzymes—such as NADPH oxidases and dual oxidases—produce ROS at a low, but regular, rate [5,6,7,8]. In low physiological concentrations, reactive species function as signaling molecules and as safeguards in the immune system against pathogens through their support of immune cell function [5,6,7].

In recent years, oxidative stress has been linked to IBD as a major etiologic factor and a possible target for future treatments [8,9,10,11,12]. This is due to the fact that patients with IBD generally have a higher oxidant burden, since the gastrointestinal tract is a large contributor of pro-oxidants in general, which has already been observed in quiescent disease [11,13,14]. In active IBD, the oxidative burden is even higher due to the infiltration of immune cells in the intestinal mucosa. In recent years, systemic biomarkers representing whole-body redox status have been proposed [15,16,17]. However, it has been difficult to establish whether these biomarkers are causally associated, or merely collateral markers of the disease, and what particular redox-regulated processes these biomarkers represent.

Until now, biomarkers or measuring instruments that reflect local oxidative stress have been limited. Measurement of the oxidation-reduction potential (ORP) in fecal water has been suggested as a potential method for quantifying oxidative stress linked to the microbiome [18]. By combining ORP with indexes such as the Metagenomic Aerotolerant Predominance Index (MAPI), a link between the microbiome and gut redox status could be established [18]. ORP is measured in millivolts (mV), and is considered a measure of the overall balance between oxidants and antioxidants, providing a global readout for oxidative stress [19]. ORP has long been used in the context of water sanitation and purification [20,21]. In the medical field, the first recorded use of ORP took place decades ago [22]. More recently, static oxidation-reduction potential (sORP) has been studied for implementation in multiple medical domains. For example, an increase in ORP has been linked to reduced semen quality and to poorer outcomes of congestive heart failure, and it has been demonstrated to be predictive of complications following pediatric cardiac surgery [19,23,24].

Interestingly, one study reported the use of ORP in fecal water to determine fecal redox status [18]. In 2018, Million et al. studied the impact of malnutrition on gut microbiota, reporting changes in bacterial composition, reduced diversity, and altered metabolism in children [18]. Furthermore, they studied whether malnutrition—and indirectly, changes to the microbiome—affected the ORP of feces. The authors hypothesized that ORP might be reduced due to higher levels of oxidative stress in patients with malnutrition and a reduced abundance of anaerobic bacteria [18,25,26,27]. In this study, a significant difference in ORP of 84.3 mV was found in feces when comparing malnourished children to healthy controls (adults and children) [18].

Similar to the case of malnutrition, IBD is also associated with increased oxidative stress and microbial disturbances in the gut [6,7]. Following the principles of ORP, we hypothesized that ORP values are lower in patients with IBD compared to healthy individuals. In this proof-of-concept study, we therefore aimed to evaluate the utility of fecal ORP measurement using fecal samples from patients with IBD and from healthy controls. Determination of fecal redox potential could be a very quick, easy, and relatively cheap method to detect disrupted intestinal redox signaling in those with IBD.

## 2. Materials and Methods

### 2.1. Study Population

In this proof-of-concept study, fecal samples of patients with CD (*n* = 5) or UC (*n* = 5), and healthy controls (*n* = 5) were selected. The patients participated in the 1000IBD project, comprising a cohort of over 1000 IBD patients from the Northern provinces of the Netherlands, who provided prospectively collected samples [28]. Healthy control samples were obtained from the LifeLines-DEEP cohort, a subcohort of the LifeLines Cohort Study, which is a large, multigenerational cohort study featuring over 167,000 participants (10%) from the northern population of the Netherlands [29]. Demographic and clinical characteristics of patients were collected, including sex; age; body mass index (BMI); the Montreal disease classification registered during endoscopic assessment within at least 12 months of sample collection; and clinical disease indices, including the Harvey Bradshaw index (HBI) for CD and the Simple Clinical Colitis Activity Index (SCCAI) for UC. For this study, we included both patients with quiescent and active disease. Patients with CD who have upper gastrointestinal disease or active perianal disease were excluded from the study. Only patients aged 18–65 years were included. For the healthy controls, only age, sex, and BMI were collected from individuals who had self-proclaimed gastrointestinal health. Furthermore, the absence of gastrointestinal conditions in healthy controls was ascertained by a licensed gastroenterologist.

### 2.2. Measurement of ORP in Fecal Samples

Samples from patients with IBD and healthy controls were obtained according to the same protocols. A package for fecal collection was sent to each of the participants’ homes. Feces were collected in non-sterile tubes and immediately frozen in a standard freezer. Mostly within two weeks, the fecal material was transported in dry ice to the hospital (University Medical Center Groningen) and stored at −80 °C until ORP analysis. The protocol for preparing the fecal samples (fecal water) was based on the method used by Million et al., albeit with minor modifications [18]. To perform the method, at least 0.4 g of fecal sample was weighed into a 15 mL Falcon tube, and a 0.1 g/mL suspension was prepared with distilled water and vortexed until a homogeneous solution was obtained. The environmental conditions of all samples were kept constant during measurements, to avoid shifts in the readout due to temperature-dependent pH and redox potential. For this reason, all steps except the weighing and centrifugation of fecal samples were performed within a biosafety class II cabinet. Materials required included Gibco distilled water; a pH electrode especially for soil, mud, and slurry; redox test solutions; calibration solutions pH = 4, pH = 7, and pH = 10; 15 mL Falcon tubes; pipettes; vortex; and 70% EtOH.

The PCE-228 redox and pH meter equipped with the redox electrode (PCE Holding GmbH, Hamburg, Germany) was used to measure the ORP value of all samples, and was calibrated daily before performing measurements. This ORP electrode uses a platinum/gold sensing electrode with a Ag/AgCl reference junction, which makes it possible to provide and receive electrons from the solution that is being measured [30]. This results in a positive ORP value when the probe provides electrons (oxidation), and a negative ORP value when the probe receives electrons (reduction). The redox electrode was flushed with distilled water and then rinsed in a dedicated Falcon tube with 5 mL of distilled water after each measurement. Redox test solutions were measured for function tests, and the mV of all samples was measured and manually recorded. The ORP was measured for 3 min (timepoint 1). The suspension was then centrifuged at 4000× *g* for 10 min at room temperature, and the supernatant was transferred into a new Falcon tube. Appropriate aerosol-tight covers were used during centrifugation. After this, the ORP was measured again for 3 min (timepoint 2). After the second measurement, the redox electrode was rinsed, disinfected, and stored in the Electrode Storage Solution.

### 2.3. Stability of ORP Measurements across Various Solutions

In a second experiment, we aimed to test the impact of time on ORP. Four solutions (tap water, distilled water, alkaline water (200 mg/mL), and green tea) were measured at nine timepoints of five minutes each. The ORP was measured after each round of five minutes, followed by a pause period of 15 min before the next measurement would start. The aforementioned solutions were selected with the aim of obtaining a wide variety of ORP values. The expected ORP values were based on those from previous experiments, in which the ORP of tap water (+275 mV), distilled water (+220 mV), alkaline water (200 mg/mL SODA; −20 mV), and green tea (+147 mV) were measured, according to previous literature [31]. The environmental conditions of all samples were kept constant during measurements, to avoid shifts in ORP values that might be attributed to temperature-dependent pH changes. Once again, the PCE-228 redox and pH meter equipped with the redox electrode was used for these measurements. ‘Stable’ measurements were defined as ORP values deviating less than 20 mV, or <15%, between measurements.

### 2.4. Statistical Analysis

The demographic and clinical characteristics of the study participants were presented as medians (interquartile range (IQR)), or as proportions (*n*) with corresponding percentages (%). We used a Wilcoxon signed-rank test to compare ORP between timepoints 1 and 2. For comparisons between the three different groups, the Kruskal–Wallis test was used. Two-tailed *p*-values ≤ 0.05 were considered statistically significant. Data analysis was performed using R (version 4.3.0, Vienna, Austria), and data visualization was performed using RStudio (version: 2022.12.0.353, JJ Allaire, Boston, MA, USA)

### 2.5. Ethical Considerations

All participants provided written informed consent prior to sample collection. This study was approved by the Institutional Review Board (IRB) of the UMCG, Groningen, the Netherlands (in Dutch: “Medisch Ethische Toetsingscommissie”, METc; IRB no. 2008/338), and was conducted in accordance with the principles of the Declaration of Helsinki (2013). In addition, control samples were used from the LifeLines-DEEP cohort study [29]. The LifeLines-DEEP cohort study was also approved by the IRB of the UMCG (IRB no. M12.113965) and registered at the LifeLines Research Site in Groningen.

## 3. Results

### 3.1. Baseline Characteristics

In this proof-of-concept study, ten patients with IBD (five patients with UC and five with CD) and five healthy controls were included. Six patients with IBD were female and four were male, whereas three healthy controls were female and two were male. Age was slightly different between all groups (medians 52.0 vs. 44.0 vs. 60.0 years, *p* = 0.046) (Table 1).

### 3.2. ORP Measurements Show Poor Temporal Stability across Various Test Solutions

**Figure 1:**
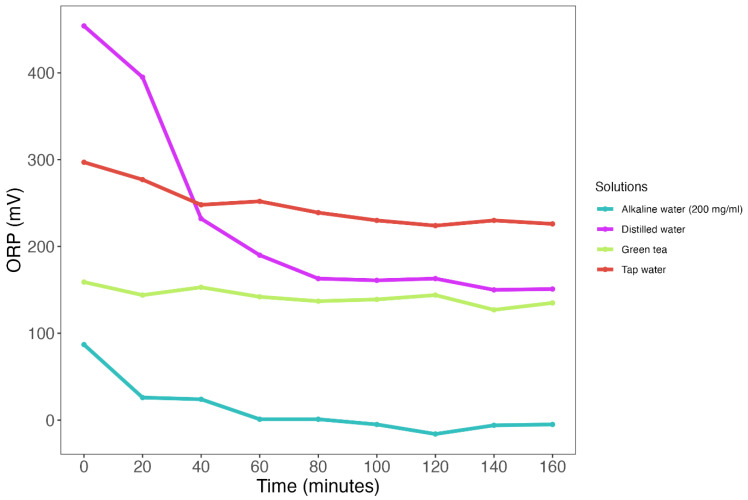
Repeated ORP measurements across four different test solutions. Solutions include distilled water (purple line), tap water (red line), green tea (green line) and alkaline water (blue line). Each measurement was performed for 5 min followed by a 15 min stabilization period.

Subsequently, we measured ORP values across four different test solutions with different expected ORP values, which appeared to vary quite extensively (Figure 1, Table S1). Three solutions showed a decrease in ORP over time: tap water (median: +239 mV [IQR: 230–252]) decreased by 71 mV (−24%); distilled water (median: +163 mV [IQR: 161–232]) decreased by 303 mV (−67%); and alkaline water (median: +1 [IQR: −5–24]) decreased by 93 mV (−106%). Only green tea (median: +142 mV [IQR: 137–144]) was considered ‘stable’ with a decrease in ORP of 24 mV (−15%) (Figure 1, Table S1).

### 3.3. Both Patients and Controls Exhibit Low Redox Potential Variability

**Figure 2:**
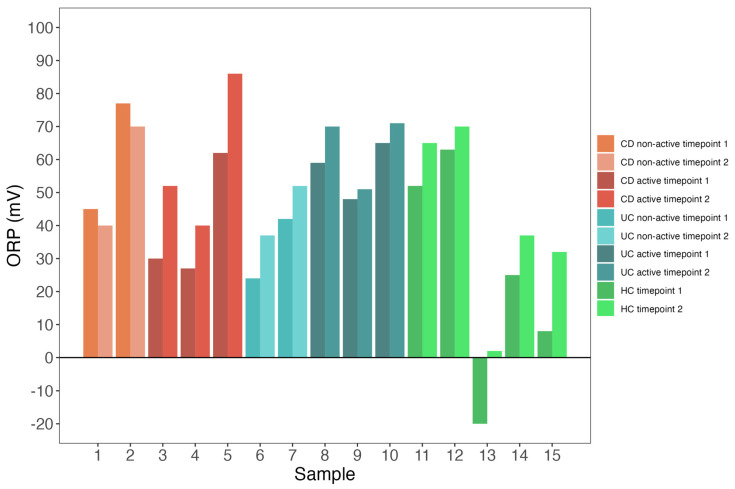
Both patients with IBD and controls exhibit low redox potential variability. Bars indicate ORP values across two timepoints for each individual, separately. Numbers on the *x*-axis represent individual participants.

**Figure 3:**
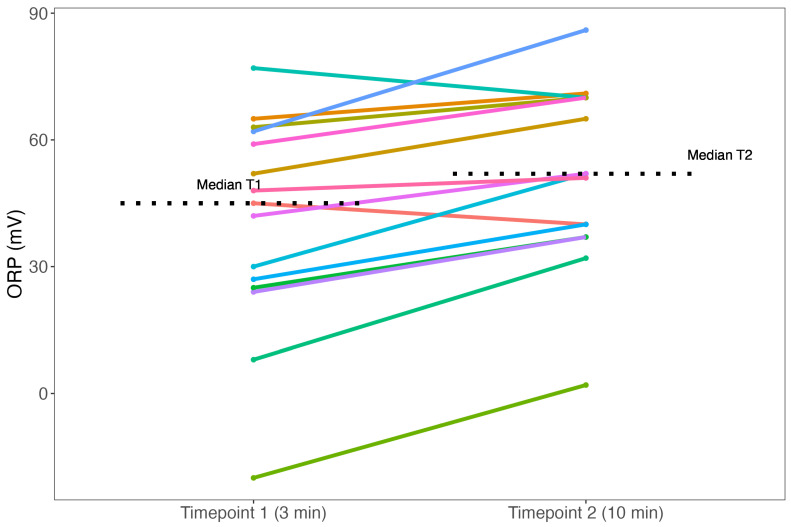
Individual changes in ORP values between two separate timepoints. The 15 colored lines represent the 15 individuals and the ORP values at timepoint 1 (3 min) and timepoint 2 (10 min). The black dotted lines are the medians of timepoints 1 and 2. Abbreviations: ORP = oxidation-reduction potential; T1 = timepoint 1; T2 = timepoint 2.

Among the seven individuals, redox potential variability was low at timepoint 1 (median: +45 mV [IQR: 26–61]) (Figure 2). Redox potential variability remained low at timepoint 2 (median: +52 mV [IQR: 39–70]) (Figure 2). Furthermore, individual changes in ORP varied between timepoint 1 and timepoint 2. In ten individuals, ORP increased (minimum of +3 mV, maximum of +24 mV) (Figure 3). In two individuals, ORP decreased by 5 mV and 7 mV, respectively (Figure 3).

## 4. Discussion

In this study, we demonstrated that measurements of ORP are characterized by poor temporal stability—showing high fluctuations over time—and low variability when comparing measurements taken from the fecal samples of patients with active and non-active IBD, and when comparing measurements taken from IBD patients with those from healthy controls. These findings suggest that ORP measurements may not be suitable for the quantification of fecal redox status in IBD. Although this proof-of-concept study included only a few samples and, thus, low sample size was at least partially responsible for the absence of a difference between patients and controls, the high degree of temporal fluctuations is likely to exceed that of potential inter-individual variability. Across all test solutions used to evaluate temporal patterns in fecal redox potential, we observed a general decline in ORP values. We theorize that these test solutions might have consumed their redox potential, resulting in more stable ORP values over time. This observation of slow stabilization (up to several hours) of ORP may contribute to the absence of differences between patients with IBD and controls, which in turn would decrease the potential clinical utility of such measurements.

Unfortunately, we were not able to replicate the results of the previous study reported by Million et al., who examined ORP values in fecal samples from malnourished individuals and healthy controls [18]. Although this study did not specifically focus on patients with IBD, both gut redox status and microbiome composition showed distinct alterations in those afflicted with this disease, including a general decrease in the abundance of commensal anaerobic bacteria in the stool [5,11]. Therefore, the absence of a clear difference between patients and controls led us to reject our initial hypothesis. The largest discrepancy between our data and the results by Million and colleagues was observed in the healthy control samples [18]. Million et al. reported ORP values below −100 mV in fecal samples [18]. In the present study, however, only a few small negative ORP values were detected in alkaline water, with a minimum value of −16 mV. Only one healthy control showed a negative value upon the first ORP measurement. Overall, the healthy controls seemed to have a slightly lower ORP compared to patients with IBD, although this was not statistically significant. Furthermore, the differences in ORP between patients and controls at timepoint 1 (23 mV (CD), 20 mV (UC)) and timepoint 2 (15 mV (CD), 15 mV (UC)) were not comparable to the differences found by Million et al., since most of those differences pertained to values exceeding 100 mV [18]. To check whether the ORP measurement probe could measure negative ORP, we therefore tested a sodium hydroxide [NaOH] solution with a pH of 14. Although pH is not the only factor that influences ORP, it is considered a substantial contributing factor. This NaOH solution had an ORP of −70 mV. Although the manufacturer of the probe reports that measurement values might differ between probes, we strongly doubt that the observed differences can only be explained by this inter-probe variability, especially because the positive ORP measurements reported by Million et al. were either similar to or higher than those in our study [18]. 

A recent study performed by Pham et al. investigated the effects of colon-targeted vitamins on the composition and metabolic activity of the human gut microbiome [27]. Here, the investigators also studied ORP values in healthy participants, and the effects of vitamin C and placebos on ORP measurement values. The net effect of vitamin C on ORP was −/+ 5 mV, which, along with the values reported in previous studies, is almost negligible. However, these differences were similar to our measured ORP values in fecal samples, with the difference between medians of timepoints being 11 mV (Figure 2). This suggests that ORP values generally do not exhibit extensive variation. This aspect, along with the inherent instability observed in ORP measurements, renders it unsuitable for implementation as a diagnostic tool or a tool for monitoring treatment effects. In addition, similar to the observations of Pham et al. (unpublished data), we only observed very few instances of negative ORP.

The investigation of ORP in fecal samples from patients with IBD is an intriguing area of research due to the potential implications for disease diagnosis and treatment. Established biomarkers of intestinal inflammation, such as fecal calprotectin, lipocalin-2, and neutrophil-derived elastase are more indirectly linked to oxidative stress. These biomarkers provide a rather indirect reflection of the oxidative stress burden, primarily due to the substantial contribution of ROS overproduction for neutrophil-derived NADPH oxidases [3,4,5,6]. However, these biomarkers do not adequately capture microbiome-related redox status in the intestines. In this regard, ORP measurements of fecal water could theoretically serve as a means to address this gap. However, currently available techniques for measuring (fecal) redox status, such as Ferric Reducing Antioxidant Power (FRAP) assay and 2′2′-diphenyl-1-picrylhydrazyl (DPPH) assay, are both accurate and validated [32,33,34]. Notably, however, these methods are also inherently complex and costly, whereas ORP measurements in fecal samples could serve as a rapid and cost-effective alternative. Our study revealed that ORP measurements exhibited significant temporal instability and did not show significant variations between patients with IBD and healthy controls.

Therefore, our study suggests that ORP measurements of fecal samples from patients with IBD are unsuitable for the accurate measurement of the redox status of fecal water.

Strengths of this study include the incorporation of samples from both patients and controls. Furthermore, the implementation of lengthy measurements (lasting for five minutes) and multiple timepoints in both experiments offers valuable insights into the longitudinal dynamics of ORP alterations. However, there are also certain limitations that warrant recognition. One such limitation pertains to the limited number of samples that were analyzed. It is essential to highlight that the primary objective of this study was, however, not to uncover statistically significant findings between patients with IBD and healthy controls, since achieving such significance would have, at least, required a larger sample size. Instead, the primary objective was to evaluate the utility of the ORP measurement probe within a diagnostic framework and to identify potential emerging trends. The decision to include only a limited number of samples was based on the rationale of uncovering preliminary indications, rather than drawing definitive statistical conclusions. Furthermore, the high instability of the readouts as we initially observed across the redox test solutions did not warrant further in-depth analysis for statistical power.

## 5. Conclusions

In conclusion, in this study, we demonstrated that fecal ORP measurements do not seem to be a reliable and suitable tool for determining fecal redox status, neither in Dutch patients with IBD nor in healthy controls. Furthermore, we did not find significant differences between the ORP values of the fecal samples from patients with IBD and those from healthy controls. Although comparable methods were used regarding the preparation and measurements of ORP in fecal samples, as previously described, we were not able to replicate these earlier results [18]. Taken together, based on our findings, we do not recommend that the scientific community further pursues the investigation of the ORP redox probe used in this study for the determination of fecal redox status. However, if new probes were to be specifically designed and manufactured for this purpose, accompanied by thorough investigation of the instability of ORP in fecal samples and a better understanding of the local redox status of patients with IBD, ORP measurements could potentially serve as a time- and cost-effective diagnostic tool.

## Figures and Tables

**Table 1:** Baseline characteristics of study participants.

Characteristic	CD, N = 5 ^1^	UC, N = 5 ^1^	HC, N = 5 ^1^	*p*-Value ^2^
ORP Timepoint 1	45.0 (30.0, 62.0)	48.0 (42.0, 59.0)	25.0 (8.0, 52.0)	0.47
ORP Timepoint 2	52.0 (40.0, 70.0)	52.0 (51.0, 70.0)	37.0 (32.0, 65.0)	0.36
Sex				1.00
Female	3 (60%)	3 (60%)	3 (60%)	
Male	2 (40%)	2 (40%)	2 (40%)	
Age (years)	52.0 (48.0, 53.0)	44.0 (37.0, 45.0)	60.0 (57.0, 65.0)	0.05
Body Mass Index (kg/m^2^)	26.9 (25.9, 32.7)	24.8 (23.7, 26.8)	-	0.35
Active disease	3 (60%)	3 (60%)	-	
HBI	5 (2, 6)	-	-	
SCCAI	-	4 (2, 5)	-	
Montreal Age				
A1 (<17 y)	0 (0%)	0 (0%)	-	
A2 (17–40 y)	3 (60%)	5 (100%)	-	
A3 (>40 y)	2 (40%)	0 (0%)	-	
Montreal Location				
L1 (Terminal ileum)	1 (20%)	-	-	
L2 (Colon)	0 (0%)	-	-	
L3 (Ileocolon)	4 (80%)	-	-	
Montreal Behavior				
B1 (Non-stricturing, non-penetrating)	3 (60%)	-	-	
B2 (Stricturing)	1 (20%)	-	-	
B3 (Penetrating)	1 (20%)	-	-	
Montreal Extension				
E1 (Proctitis)	-	1 (20%)	-	
E2 (Left-sided UC)	-	0 (0%)	-	
E3 (Pancolitis UC)	-	4 (80%)	-	
Medication use				
Aminosalicylates, *n* (%)	0 (0%)	4 (80%)	-	
Steroids, *n* (%)	1 (20%)	1 (20%)	-	
Thiopurines, *n* (%)	0 (0%)	0 (0%)	-	
Anti-TNF, *n* (%)	2 (40%)	1 (20%)	-	
Surgical history, *n* (%)				
Ileocecal resection, *n* (%)	2 (40%)	0 (0%)	-	
Colectomy, *n* (%)	1 (20%)	0 (0%)	-	

Abbreviations: ORP = oxidation-reduction potential; ORP timepoint 1 = measurement after 3 min; ORP timepoint 2 = measurement after 10 min. Active disease = SCCAI > 2 or HBI > 4. CD = Crohn’s disease; UC = ulcerative colitis; HC = healthy control;.^1^ Median (IQR); *n* (%). ^2^ Kruskal–Wallis rank sum test; Fisher’s exact test.

## Data Availability

The dataset(s) used for the current study are available from the corresponding author upon reasonable request.

## Supplementary Materials

The following supporting information can be downloaded at: https://www.mdpi.com/article/10.3390/biomedicines11123107/s1. Table S1: Repeated ORP measurements at several timepoints across four different test solutions. Each measurement was done for five minutes. A pause of fifteen minutes was held between two measurements.
