# Supplementary material for: Unsuitability of the Oxidation-Reduction Potential Measurement for the Quantification of Fecal Redox Status in Inflammatory Bowel Disease
Source: Biomedicines. 2023 Nov 21;11(12):3107. doi: 10.3390/biomedicines11123107 (PMC10741202; doi:10.3390/biomedicines11123107)
Supplement: Supplementary file 1 [file biomedicines-11-03107-s001.zip › biomedicines-2664208-supplementary.pdf]

Table S1. Repeated ORP measurements at several timepoints across four different test solutions. Each measurement was done for five minutes. A pause of 15 minutes was held between two measurements.

Measurement (15 min intervals)	Tap water	Distilled water	Alkaline water (200 mg/ml)	Green tea
**1**	297	454	87	159
**2**	277	395	26	144
**3**	248	232	24	153
**4**	252	190	1	142
**5**	239	163	1	137
**6**	230	161	-5	139
**7**	224	163	-16	144
**8**	230	150	-6	127
**9**	226	151	-5	135
**Overall change in mV (%)**	71 (24%)	303 (67%)	93 (106%)	24 (15%)
